# Combining Numerical Relational and Fundamental Motor Skills to Improve Preschoolers’ Early Numeracy: A Pilot Intervention Study

**DOI:** 10.1007/s13158-022-00329-8

**Published:** 2022-05-07

**Authors:** Pinja Jylänki, Elina Sipinen, Theo Mbay, Arja Sääkslahti, Pirjo Aunio

**Affiliations:** 1 Faculty of Educational Sciences, University of Helsinki, Helsinki, Finland; 2 Faculty of Sport and Health Sciences, University of Jyväskylä, Jyväskylä, Finland

**Keywords:** Early numeracy, Fundamental motor skills, Intervention, Numerical relational skills, Preschool

## Abstract

The aim of this pilot study was to examine the immediate and long-term effects of an intervention program that aims to improve preschoolers’ (*N* = 36, *M* = 4.49 years, SD = 0.35) early numeracy skills by combining the learning of numerical relational skills via story reading with fundamental motor skill practice. The intervention program was piloted with two study designs: a within-subject repeated-measures design with 18 children (study I), and a quasi-experimental study design with 18 children (study II). Children’s early numeracy, symbolic magnitude processing, and fundamental motor skills were measured. Results demonstrated that children’s early numeracy and especially numerical relational skills improved during the intervention, and the intervention had larger effects on children’s early numeracy and numerical relational skills compared to the control period (study I) and control group (study II). Furthermore, the results from the delayed post-test demonstrated that the effects were maintained for 4.5–8 weeks after the intervention. These findings provide preliminary evidence that it is possible to support children’s early numeracy skills with combined learning of numerical relational skills via story reading and fundamental motor skills despite the socioeconomic or language background, and narrow the gap between low- and average-performing children.

## Résumé

Le but de cette étude pilote était d'examiner les effets immédiats et à long terme d'un programme d'intervention qui vise à améliorer les compétences précoces en numératie des enfants d'âge préscolaire (N = 36, M = 4.49 ans, SD = 0.35) en combinant l'apprentissage des compétences par la lecture d'histoires avec la pratique des habiletés motrices fondamentales. Le programme d'intervention a été piloté avec deux plans d'étude : un plan à mesures répétées intra-sujet avec 18 enfants (étude I) et un plan d'étude quasiexpérimental avec 18 enfants (étude II). La numératie précoce des enfants, le traitement de la magnitude symbolique et les habiletés motrices fondamentales ont été mesurés. Les résultats ont démontré que la numératie précoce des enfants et en particulier les compétences relationnelles numériques se sont améliorées au cours de l'intervention, et l'intervention a eu des effets plus importants sur la numératie précoce et les compétences relationnelles numériques des enfants par rapport à la période témoin (étude I) et au groupe témoin (étude II). De plus, les résultats après le test ont démontré que les effets se maintenaient de 4.5 à 8 semaines après l'intervention. Ces résultats fournissent des preuves préliminaires qu'il est possible de soutenir les compétences précoces en numératie des enfants avec l'apprentissage combiné des compétences relationnelles numériques par la lecture d'histoires et les habiletés motrices fondamentales malgré le contexte socio-économique ou linguistique, et de réduire l'écart entre les enfants peu performants et les enfants moyenne.

## Resumen

El objetivo de este estudio piloto fue examinar los efectos inmediatos y a largo plazo de un programa de intervención educativa. Dicho programa busca mejorar las habilidades numéricas tempranas de niños en edad prescolares (N = 36, M = 4.49 años, SD = 0.35). Mediante de la combinación del aprendizaje de habilidades de relación numérica a través de la lectura de cuentos con la práctica de habilidades motoras fundamentales. El programa de intervención se ha pilotado con dos diseños de estudio distintos: un estudio de observación y medidas reiteradas de un mismo sujeto, con 18 niños (estudio I); y un estudio cuasiexperimental con otros 18 niños (estudio II). En ellos se midieron las habilidades numéricas tempranas, el procesamiento de magnitudes simbólicas y las habilidades motoras fundamentales. Los resultados han demostrado que durante la intervención, y en comparación con el periodo de control (estudio I) y el grupo de control (estudio II), se han mejorado las habilidades numéricas tempranas de los niños y, en especial, las habilidades de relación numérica. Además, los resultados de la evaluación posterior a la intervención demostraron que los efectos se mantuvieron durante 4.5 - 8 semanas después de la intervención. Estos resultados aportan una evidencia preliminar que defiende la posibilidad de mejorar las habilidades numéricas tempranas de los niños a través del trabajo de las mismas con lectura de cuentos y el desarrollo de funciones motoras fundamentales. Todo ello a pesar del entorno socioeconómico o lingüístico del niño; lo que permite reducir las diferencias entre los niños de bajo y medio rendimiento.

## Introduction

Previous research has shown that individual differences in children’s early numeracy (EN) can be observed already before formal schooling begins (Aunio et al., 2015), and the achievement gap between low- and high-performers widens during the preschool years (i.e., from years 3 to 6; Anders et al., 2012). This highlights the necessity for developing effective intervention programs to support low-performing preschoolers, which can prevent them from being at risk of later mathematical learning difficulties (Aunio et al., 2015).

Recommendations for improving EN in the early years include combining EN learning with other curriculum areas and exploring EN through different contexts; for instance, joint story reading offers a valuable opportunity to initiate mathematical discussions with children (Education Endowment Foundation, 2020) and offers meaningful way to learn EN (Van den Heuvel-Panhuizen & Elia, 2011). Positive effects of a story reading intervention on children’s numerical relational skills—which are central in the development of EN (Toll & Van Luit, 2014)—have been observed (Hassinger-Das et al., 2015). In addition to story reading, activities which allow children to be physically active may support children’s learning (Jylänki et al., 2022). Children learn naturally through play (Whitebread et al., 2009), and can be seen as active players, since their play usually includes bodily movements instead of passive observing (Thompson & Goldstein, 2020). However, due to activities that require sitting (Määttä et al., 2019), children’s possibilities to be physically active and use bodily movements are often limited in preschools (Vanderloo et al., 2013).

Bodily movements, operationalized often as fundamental motor skills (FMS), have been associated with EN in preschool-aged children (Gashaj et al., 2019). Intervention studies, which combined EN and FMS practice, have found positive (Fischer et al., 2011; Shoval et al., 2018), and even long-term effects on EN performance (Beck et al., 2016). In addition, interventions which combine FMS with the learning of academic or cognitive skills have been found effective in preschoolers, and combined interventions may even be superior compared to learning academic or cognitive skills alone (Jylänki et al., 2022). One possible explanation for the close association between FMS and the learning of academic and cognitive skills is the fact that especially complex FMS tasks activate the same brain areas as complex cognitive tasks, resulting in a co-activation of these brain areas (Diamond, 2000). Furthermore, these effects may be mediated through an increase in executive functions—inhibition in particular—following FMS practice, which might then be reflected as improved cognitive and academic skills (e.g., ability to inhibit irrelevant information during given tasks; Chan et al., 2022; Pesce et al., 2021). In light of previous research, it is plausible that combining the learning of EN with FMS practice may result in long-term improvements in EN. Our pilot study examined the immediate and long-term effects of a Movement with Early Numeracy (MovEN) intervention program that aims to improve EN by combining the learning of numerical relational skills via story reading with FMS practice.

### Preschoolers’ Early Numeracy

In 3- to 4 year-old children, relevant EN skills include numerical relational, counting and symbolic magnitude processing skills (SYMP; Clements & Sarama, 2009; Litkowski et al., 2020). The understanding of numerical relational skills in particular has shown to be critical for later numeracy development (Purpura et al., 2011; Toll & Van Luit, 2014). Numerical relational skills consist of concepts that are used to describe quantity relations (e.g., more, half) and spatial relations (e.g., above, between; Toll & Van Luit, 2014) between entities. Studies with preschoolers have demonstrated that the learning of numerical relational skills is important from early on (Purpura et al., 2011) and that children with mathematical learning difficulties in particular benefit from intensified numerical relational skill practice (Hassinger-Das et al., 2015).

EN and language skills have found to be developmentally related (Purpura et. al., 2011), and learning numerical concepts is an important shared area within this relationship (Toll & Van Luit, 2014). In order to learn numerical relational skills, children are required to possess adequate language skills to understand these linguistically expressed concepts (Purpura & Lonigan, 2013), and they need to understand the numerical information embedded in the tasks (Toll & Van Luit, 2014).

Discussion-based approaches that include discussion and instruction following reflection (Gifford, 2005), such as dialogic story reading where the teacher uses questions and prompts children for discussion, have shown to be effective for improving both EN and language skills in preschoolers (Purpura et al., 2017). Indeed, story reading interventions have been found to be an effective way to increase different areas of numerical relational skills knowledge, such as concepts related to counting and number operations (Hassinger-Das et al., 2015), length measurement (Van den Heuvel-Panhuizen & Elia, 2011), and geometry (Casey et al., 2008). Furthermore, Casey and her colleagues (2008) demonstrated that story reading intervention was more effective compared to intervention without the story reading component, and Purpura and his colleagues (2017) showed that story reading intervention improved not only numerical relational skills, but also children’s EN. These findings suggest that the use of story reading is an effective way to increase preschoolers’ numerical relational skills as well as EN.

### Early Numeracy and Fundamental Motor Skills

In recent decades, studies examining the effects of physical activity (including FMS) on cognitive skills have increased rapidly (Pesce et al., 2021). Previous studies have mainly focused on school-aged children, while children in their early years remain understudied (Pesce et al., 2021). Studies have shown that FMS (balance, manipulative and locomotor movement skills; Donnelly et al., 2017) are associated with EN in preschoolers (Gashaj et al., 2019). Both FMS and EN skills start to develop at the age of two to three years (Clements & Sarama, 2009; Gallahue & Ozmun, 2002), and when children are 4- to 5-year old, their skills are more developed and the gap between the low- and high-performers starts to broaden (Anders et al., 2012; Starkey & Klein, 2008). Thus, 4-to-5-year-olds are a developmentally desirable group to be supported with an intervention. Previous intervention studies that combined FMS with EN learning (Table 1) have demonstrated positive effects on EN (Fischer et al., 2011; Shoval et al., 2018), and the effects have been larger than in EN interventions without an FMS component (Fischer et al., 2011).

**Table 1:** Previous combined movement and EN interventions in preschoolers

Reference	Study design	Sample	Outcome measure	Exposure	Intervention	Results	Delayed effects
(Fischer et al., 2011)	CCT	*N* = 22; 5.8 years	Number line estimation task and TEDI-MATH (i.e., counting principles, object counting, Arabic digits, number words and calculation)	Three 10- to 15 min training sessions within 3 weeks	A number magnitude comparison tasks on a dance mat. Children received training individually	Greater improvements in number line estimator and counting principles subtests compared to tablet PC intervention	Not measured
(Shoval et al., 2018)	CCT	*N* = 106; 4.9 ± 0.58 years	Mathematics achievement test (i.e., quantitative concepts, understanding sequence, counting, enumerating, conservation of quantities and number permanence, verbal reasoning problems) and sequencing test of ordinal numbers	Five times a week, 90 min outdoor and 90 min indoor activities for 145 days	"Mindful movement" was integrated with academic learning (science, mathematics, reading and writing) in outdoor and indoor learning environments	Intervention led to the greater improvements in the math perforsmance compared to BAU	Not measured
(St. Laurent et al., 2018)	CCT	*N* = 52; 4.1 ± 0.1 years	Number recognition (i.e., numbers from 1 to 15 in random order)	Two to three times a week, total of 10 to 35 min training, for 12 weeks	Active learning lessons (5–10 min) 2–3 times a week. These were placed with FMS training (30 min) every other week. Video PA breaks (5 min) three times a week	No effects	Not measured

*CCT* Controlled clinical trial; *BAU* Business-as-usual control group; *PC* Personal computer

Although the previous combined FMS and EN interventions have been found effective, there are some apparent limitations. For instance, some shortcomings were found in the intervention designs: the long-term effects were not assessed (Fischer et al., 2011; Shoval et al., 2018; St. Laurent et al., 2018) or narrow outcome measures were used that only measured one dimension of EN (e.g., number recognition; St. Laurent et al., 2018). The aforementioned limitations, and the fact that there are only a few studies assessing the effects of combined FMS and EN interventions in preschoolers, underline the necessity to investigate the effects of intervention programs that support the development of 4-to-5-year-olds’ FMS and EN skills and possibly narrow the gap between the low- and high-performers.

### Present Study

Research in school-aged children has shown a correlative relationship between FMS and EN learning (Macdonald et al., 2018), as well as demonstrated the superiority of combined FMS and EN interventions in comparison with EN learning alone (Have et al., 2018). However, the effects in preschoolers remain understudied (Jylänki et al., 2022). This is crucial, since it has been shown that individual differences can be observed already before formal schooling begins, and the importance of early support for low-performers is well demonstrated (Aunio et al., 2015). In addition, while previous EN interventions have been found effective, long-term effects have seldom been found nor reported (Aunio, 2019). Thus, the aim of the present study was to examine the immediate and long-term effects of the MovEN-intervention that aims to improve EN by combining the learning of numerical relational skills via story reading with FMS practice. The effects of the MovEN-intervention on 4 year-old children’s EN performance were examined with two study designs. First, a within-subject repeated-measures design (study I) was conducted with two intervention groups, and then, a quasi-experimental design with an intervention and an average performance control group (study II) was carried out to verify the findings from study I.

## Methods

### Participants

A total of 36 children (*M* = 4.49 years, SD = 0.35); 21 girls and 15 boys, from four preschools in the metropolitan area, participated in this pilot study. Of these, 18 children participated in study I, and 18 children (9 in the intervention and 9 in the average performance control group) in study II. Preschools participating in a larger longitudinal project (*N* = 21, Active Early Numeracy) were invited to participate in the MovEN-intervention, of which four preschools volunteered to participate. Children in the intervention groups were, at first, identified as at risk in numerical relational skills learning based on their teachers’ experience, and secondly measured with the EN test (i.e., children who were more than one standard deviation below the average in the numerical relational skill measures were regarded as low-performing). Children’s parental consent, as well as the university ethics approval (August 28, 2019), was obtained prior to the start of the study. Participation was voluntary, and withdrawal from the study was possible at any time.

Participating children had heterogeneous language and socioeconomic backgrounds, and thus both language skills and socioeconomic status (SES) were used as underlying variables. Children’s guardians filled in a questionnaire concerning the family’s SES. Participant characteristics and SES are presented in Table 2. Study I participants’ guardians displayed a greater representation of higher education and a lower degree of unemployment compared to the SES factor distribution in the respective municipality, while the household net income was in line with the municipality’s average. In study II, the education and employment background of the control group followed a similar pattern, apart from demonstrating a greater net income in comparison with the average of the municipality. In the intervention group, the participant’s families had an overrepresentation of lower education (i.e., high school) and unemployment, despite having greater average net income compared to the municipality. The first language of the participants in study I and study II control group was Finnish, while in study II intervention group, nearly half of the families reported other than Finnish as their first language, which is an overrepresentation compared to the municipality’s average. However, it should be noted that all of the families did not return the questionnaire concerning the family’s SES (i.e., 83% of the families returned the questionnaire), possibly influencing the results.

**Table 2:** Baseline characteristics and socioeconomic status of the participants

	Study I		Study II			
			Intervention		Control	
	*M*	SD	*M*	SD	*M*	SD
Age (years)	4.39	0.44	4.57	0.25	4.61	0.14
Weight (kg)	19.66	2.67	19.83	2.46	19.60	2.39
Height (cm)	109.25	5.17	106.83	2.26	110.89	2.56

	*n*	%	*n*	%	*n*	%
*Gender*						
Female	11	61	4	44	6	67
Male	7	39	5	56	3	33
Total	18		9		9	
*Home language*						
Finnish	14	100	4	57	9	100
Other	0	0	3	43	0	0
Total	14		7		9	
*Guardian 1 educational background*						
Master’s degree	8	57	1	14	7	78
Bachelor’s degree	4	29	2	29	2	22
High school or vocational degree	2	14	3	43	0	0
Primary school or no degree	0	0	1	14	0	0
Total	14		7		9	
*Guardian 1 employment status*						
Unemployed	0	0	2	29	0	0
Studying	1	7	1	14	0	0
Part-time employed	1	7	0	0	1	11
Full-time employed	12	86	4	57	8	89
Total	14		7		9	
*Combined net income (euros/year)*						
Less than 19,999	0	0	1	14	0	0
20,000–39,999	1	8	0	0	0	0
40,000–59,999	7	58	1	14	2	22
60,000–79,999	2	17	4	58	2	22
More than 80,000	2	17	1	14	5	56
Total	12		7		9	

Children’s language skills were measured before the intervention with receptive and expressive language items from Lene (Valtonen & Mustonen, 2007; Valtonen et al., 2004). The items varied for age groups and included items assessing the understanding of instructions and receptive questions. The test consisted of eight and eleven items with a maximum score of 12 and 19 for 3-, and 4 year-old children, respectively. In study I, language skills were measured, on average, 7 months before the intervention, while in study II language skills were measured at the same time as outcome measures. The test demonstrated good reliability in the total sample of 4- (*α* = 0.81, *N* = 140) and 3 year-old children (*α* = 0.79, *N* = 141) in the longitudinal Active Early Numeracy-project.

Children’s language skills (receptive and expressive language, Valtonen & Mustonen, 2007) were compared to the corresponding reference group (i.e., children in the longitudinal project; *N* = 365) prior to the intervention (Table 3). In study I, 4 year-olds’ language skills were in line with the reference group, while 3 year-olds’ language skills were significantly better compared to the reference group. In study II, the intervention group had significantly lower language skills compared to the reference group, whereas the average performance control group’s language skills were in line with the reference group.

**Table 3:** Children language skills compared to the corresponding reference group

	Children	Language skills				
	*n*	*M*	SD	*U*	*z*	p*
*Study I*						
3 year-olds	13	11	1.3	390.0	− 3.288	**0.001**
4 year-olds	5	18	1.4	335.5	− 0.917	0.359
*Study II*						
Intervention	9	14.9	4.6	239.0	− 2.197	**0.028**
Control	9	18.2	1.0	315.0	− 1.269	0.204
*Reference group*						
3 year-olds	127	9.6	2.0			
4* year-olds*						
Study I	175	16.8	3.3			
Study II	93	17.3	2.4			

Bolded *p*-values are statistically significant (*p* < 0.05)

*Compared to the corresponding reference group

In study II, independent samples *t* test was conducted to reveal differences between the intervention and average performance control group prior to the intervention. There was no statistically significant differences between the intervention and average performance control groups in EN total score *t*(11.721) = 2.09, *p* = 0.060, counting tasks *t*(10.105) =  − 0.45, *p* = 0.661, SYMP *t*(10.199) =  − 0.89, *p* = 0.394, and FMS *t*(13.737) = 0.27, *p* = 0.788 prior to the study. However, the average performance control group had significantly greater numerical relational scores compared to the intervention group before the intervention, *t*(12.447) = 4.19, *p* = 0.001. In addition, significant differences between the intervention (*M* = 14.9, SD = 4.6) and the average performance group (*M* = 18.2, SD = 1.0) in language tasks, *U* = 15.5, *z* =  − 2.27, *p* = 0.023, were detected prior to the intervention; favoring the average performance group. There was also a significant correlation between language skills and ENT results at baseline (*r* = 0.476, *p* = 0.046), suggesting that children with greater language skills also demonstrated superior EN skills.

### Study Design

**Fig. 1:**
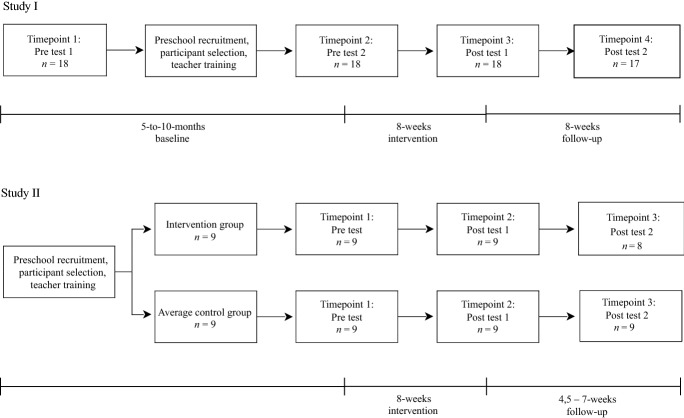
Intervention flow diagram

The effects of the MovEN-intervention were examined with two study designs. In study I (*n* = 18), a within-subject repeated-measures design was used because the control group was not available due to COVID-pandemic-related restrictions in preschools. Children’s EN and FMS were measured twice before and after the intervention (Fig. 1). The first two measurement points (pre 1 and pre 2) formed the baseline (i.e., 5 to 10 months), the third measurement point (post 1) was conducted immediately after the intervention, and the delayed post-test (post 2) eight weeks after the intervention. During the baseline, children followed a regular preschool curriculum (City of Helsinki Education Division, 2019) and the baseline was used as a control period for the intervention (i.e., business-as-usual).

To verify the findings from study I, we conducted study II (*n* = 18), where a quasi-experimental design was used with a MovEN-intervention group (*n* = 9) and an average performance control group (*n* = 9). Both groups were measured before, immediately after and 4.5–7 weeks after the MovEN-intervention. During the 8 week intervention period, the average performance control group followed a regular preschool curriculum (City of Helsinki & Education Division, 2019).

### Outcome Measures

#### Early Numeracy

EN was measured with the Early Numeracy Test (ENT; Van Luit et al., 2006), which includes 40 items that measure children’s numerical relational and counting skills. During the test, children were asked to point out the right answer, count objects or conduct paper-and-pencil tasks. The maximum score for EN was 40 points; numerical relational and counting skills each accounted for 20 points. ENT demonstrated good reliability *α* = 0.891 (*N* = 175) in the whole sample of 4 year-olds in Active Early Numeracy-project.

#### Symbolic Magnitude Processing

SYMP was measured with a one-digit subtest from the paper-and-pencil test of Symbolic Magnitude Processing (SYMP Test; Brankaer et al., 2017), which includes 60 one-digit pairs presented randomly in four columns. Digits ranged from 1 to 9. Children were asked to point out the larger number, and the researcher crossed out the number. Children had three minutes to solve as many items as possible. Before starting the test, children had four practice trials to ensure that they understood the task. A maximum of 60 points could be obtained from the SYMP Test. The sum score was used in the analysis. The SYMP test demonstrated good reliability *α* = 0.898 (*N* = 175) in the whole sample of 4 year-olds in Active Early Numeracy-project.

#### Fundamental Motor Skills

FMS were measured with a jumping sideways task from the KTK-test battery (Kiphard & Schilling, 2007). Children were asked to jump sideways over a wooden bar (60 × 4 × 2 cm). The jumping area was limited to 100 × 60 cm. Children had 15 s to perform the task, and only correct jumps (i.e., inside the jumping area and toes pointing forward) were counted. Task was repeated twice. Before the actual test, children had five practice jumps to ensure that they understood the task. Sum score of the two performances was used in the analysis.

### Intervention

The Movement with Early Numeracy (MovEN)-intervention program was developed based on the current scientific knowledge (Aunio, 2019; Gallahue & Ozmun, 2002; Hassinger-Das et al., 2015). The purpose of the MovEN-intervention was to improve children’s numerical relational skills via story reading and discussion about numerical relational concepts, combined with comprehensive FMS practice. The intervention sessions were 45 min in duration, held twice a week for eight weeks. All sessions followed the same structure (Table 4).

**Table 4:** Intervention structure

Activity	Book session	Content	Time
Beginning	All book sessions	Overview of the lesson plan with pictures	5 min
		Familiarization game with ball	
Story reading	Book session 1	Story is read for the first time with supporting questions	20 min
	Book session 2	Story is read for the second time highlighting the numerical relational words. Numerical relational words are explained to children	
	Book session 3	Children are telling the story. Words are explained by the children	
		One mathematical activity, where children uses the learned words	
Motor skill activity	All book sessions	Starts with a warm-up activity	15 min
		Following a motor skill activity combined with the numerical relational skills used in the storybook	
	Book session 1	Locomotor skills, e.g., running, jumping, hopping, galloping, rolling, leaping and dodging, horizontal jumping, sliding	
	Book session 2	Manipulative skills, e.g., throwing, catching, kicking, striking and trapping, dribbling, overhand throwing, and underhand rolling	
	Book session 3	Combined locomotor, manipulative and balance skills, e.g., obstacle course	
Conclusion	All book sessions	Recap of the learned words	5 min
		Children get to choose a sticker	
		Relaxation with music	
Total			45 min

The MovEN-intervention program included both numerical relational skills and FMS practice. Numerical relational skill practices were designed based on a previous storybook intervention (Hassinger-Das et al., 2015) and included in the beginning of each session. Over the intervention period, five general children’s storybooks with rich mathematical language expressing numerical relational skills (Table 5) were read using a dialogic reading technique (Beck & McKeown, 2001). For example, in the “Who eats first” book, a group of animals are solving who get to eat the peach. Animals are comparing themselves based on the length, weight, size of the mouth, and length of the tail. Finally, the worm gets to eat the peach, as it is the *shortest*, *lightest*, has the *smallest* mouth and *shortest* tail. During the reading session, children were encouraged to discuss about the measuring options, comparing animals and using the numerical relational concepts based on the questions that were included in the books. A particular storybook was read to completion in three consecutive sessions, before commencing to the next book. During the intervention sessions, children also conducted FMS exercises (i.e., balance, manipulative and locomotor movement skills) that included the same numerical relations as the books (e.g., throw *small* balls to *the nearest* target and *large* balls to the *farthest* goal, in which goal are *more* balls at the end?).

**Table 5:** Storybooks used in the intervention

Title	Lesson numbers in study I	Lesson numbers in study II	Author	Translator (finnish)	Numerical relational skills
Kolmikon Hedelmähetki [Trio Having a Fruit Session]	2—4^a^	2—4^a^	Maria Nilsson Thore (2019)	Raija Rintamäki (2019)	Whole/Part, Divide, Equal, Half, Same
Valtteri ja Violetti Väriliitu [Harold and the Purple Crayon]	5—7	Not used	Crockett Johnson (1955)	Riitta Oittinen (1999)	Straight/Curvy, Above/Below, Bigger/Smaller, Up/Down, More than/Less than, None
Kuka Saa Persikan? [Who Eats First?]	8—10	5—7	Ae-hae Yoon & Hae-won Yang (2012)	Rauha Sirola (2012)	Big/Bigger/Biggest, Smallest, Heavy/Heavier/Heaviest, Lightest, Long/Longer/Longest, Shortest
Mur ja Tähti [Mur and the Star]	11—13	8—10	Kaisa Happonen & Anne Vasko (2019)	–	Nothing, Left/Right, Big/Small, Up/Down, Before/After, Last
Kalle Karhu Tahtoo Lisää [Bear Wants More]	14—16	11—13	Wilson Karma & Jane Chapman (2016)	Tuula Syvänperä (2016)	Last, Many, Big, Plenty, Enough, More
Siiri ja Hurja Hunskeli [Siiri and Fierce Hunskeli]	Not used	14—16	Tiina Nopola (2008)	–	Plenty/Not at all, Above/Belov, Near, Small/Medium/Big, Same, Round, One, Two, Pair, Paired, Behind/In front

^a^The first lesson did not include story reading

### Procedure

Twenty-one preschools from the Active Early Numeracy-project were contacted in order to identify volunteering teachers to conduct the intervention in their preschools. Due to Covid-19 restrictions, we were not able to conduct the interventions by ourselves. Five teachers volunteered to conduct the intervention since they had identified children that could benefit from the numerical relational skill practice. In study I, the intervention was carried out in a group of eleven children with two teachers, and a group of seven children with one teacher (two teachers alternated between the sessions). In study II, the intervention was carried out by one teacher and children were divided into two groups (five and four children per group).

Teachers were trained to conduct the intervention via online meetings (two 1 h meetings). Teachers were provided with strict lesson plans, which were reviewed with the research team prior to the start of the intervention. Supporting questions were included in the books in order to guide the discussion during story reading activities. Each lesson was implemented according to the lesson plans. Intervention sessions were held in rooms where children had enough space to conduct the FMS exercises (e.g., a preschool gym). Teachers filled in a logbook for each lesson and participated in meetings with the research team every other week (six 30–60 min meetings in total) to ensure intervention fidelity. Based on the teachers’ experiences during study I, some modifications (e.g., one book was changed) were made to MovEN-intervention (Table 4). The first intervention (study I) began in September 2020 and the second (study II) in March 2021.

Children’s EN, SYMP, and FMS skills were measured before and after the intervention. Children’s language skills and SES were measured only before the intervention and were used as underlying variables. EN, SYMP, and language skills were measured individually in a separate room, and FMS were measured in groups of two to three children. Trained research assistants conducted all of the measurements in preschools. In both studies, children were unaware of the experimental objectives; however, the outcome assessors were not blinded to the intervention.

### Dropouts and Intervention Attendance

There were no withdrawals or dropouts during the eight-week intervention either in study I or II. The average attendance to the MovEN-intervention sessions in study I was 14 out of 16 sessions (85%) in one preschool (*n* = 11), while the attendance to the intervention sessions could not be defined in the other preschool (*n* = 7) due to inadequate logbook completion. The average attendance in the MovEN-intervention sessions in study II was 12 out of 16 sessions (78%). Missing data occurred in some measurement points due to children not being present during the measurement day or disagreeing to perform the tests.

### Statistical Analysis

All statistical analyses were conducted with IBM SPSS Statistics 25. In study II, an independent sample *t* test, with an alpha level of 0.05, was conducted to examine the differences between the intervention and the average performance control group before the intervention. Paired samples t tests were used to establish the effects of the MovEN-intervention in both studies. Due to small sample sizes, the results were confirmed with a nonparametric Wilcoxon rank sum test. Cohen’s *d* effect sizes were calculated for all outcomes (small 0.2, medium 0.5, and large 0.8; Cohen, 1988). In order to compare the gains during the intervention and the baseline in study I, we calculated gains per month as follows:

$${\text{Gain per month during baseline}} = \frac{{\left( {{\text{pre}} 2{\text{ scores}} - {\text{pre }}1\; {\text{scores}}} \right)}}{{{\text{months between pre}} 1{\text{ and pre}} 2\; {\text{measurements}}}}$$

$${\text{Gain per month during intervention }} = \frac{{\left( {{\text{post}} 1\; {\text{scores }} - {\text{pre}} 2\; {\text{scores}}} \right)}}{{{\text{months}}\; {\text{between}}\; {\text{pre}} 2\; {\text{and}}\; {\text{post}} 1\; {\text{measurements }}}}$$

In study II, gains per month were calculated as follows:

$${\text{Gain per month}} = \frac{{\left( {{\text{post}} 1\; {\text{scores}} - {\text{pre}}\; {\text{scores}}} \right)}}{{{\text{months between pre and post}} 1\; {\text{measurements}}}}$$

The distribution of language skill results was assessed with the Shapiro–Wilk test, revealing a non-normal distribution with a negatively skewed pattern. Thus, the between-group comparisons were carried out with the Mann–Whitney *U* test. Standardized z-scores were computed for the raw language skill results to allow for the unification of the scores between 3- and 4 year-old children in study I. Pearson’s correlation coefficients were calculated between language skills at baseline and ENT.

## Results

### Results of Study I

Paired samples *t* test was used to examine the immediate and long-term effects of the MovEN-intervention (Table 6). The results demonstrated that children’s EN improved significantly both during the 5 to 10 month baseline *t*(17) = 4.198, *p* = 0.001 and the eight-week intervention *t*(17) = 6.346, *p* = 0.000. The effects of the intervention were maintained in the delayed post-test 8 weeks after the intervention. The improvement in SYMP or FMS was not significant during the baseline. However, the improvement in FMS was significant during the intervention *t*(16) = 2.601, *p* = 0.019 and the effect was sustained in the delayed post-test.

**Table 6:** Descriptive statistics and results from study I paired samples *t* test

	Pre 1		Pre 2		Post 1		Post 2		Pre 1—pre 2			Pre 2—post 1			Post 1—post 2		
	*M*	SD	*M*	SD	*M*	SD	*M*	SD	*t*(17)	*p*	Cohen’s *d*	*t*(17)	*p*	Cohen’s *d*	*t*(16)	*p*	Cohen’s *d*
EN total	8.56	4.55	12.89	6.88	18.56	6.89	19.94	7.08	4.198	**0.001**	1.282	6.346	**0.000**	1.496	0.973	0.345	0.236
EN numerical relation	7.00	3.31	9.22	4.58	13.00	3.87	13.76	4.09	4.610	**0.000**	1.087	6.159	**0.000**	1.588	0.899	0.382	0.218
EN counting	1.56	2.01	3.67	3.24	5.56	3.85	6.18	3.38	2.998	**0.008**	0.706	3.376	**0.004**	0.796	0.687	0.502	0.167
SYMP	30.67	2.85	32.13	8.41	37.63	12.75	40.53	11.74	0.633^a^	0.537	0.163	1.909^c^	0.076	0.477	2.092^a^	0.054	0.523
FMS	16.70	11.37	18.61	8.77	22.44	9.21	24.88	11.87	1.950^b^	0.084	0.617	2.601^d^	**0.019**	0.613	1.603	0.129	0.389

Bolded *p*-values are statistically significant (*p* < 0.05)

^a^*t*(15)

^b^*t*(9)

^c^*t*(14)

^d^*t*(16)

To examine whether the gain was greater during the intervention as opposed to the baseline, gains per month were compared (Table 7). The results demonstrated that children had significantly greater monthly gain in EN (*M*_gain_ = 2.12, SD = 1.39) and numerical relational skills (*M*_gain_ = 1.39, SD = 0.98) during the intervention than during the baseline period. In terms of counting skills, SYMP or FMS, no significant differences were detected in monthly gain between the baseline period and the intervention.

**Table 7:** Gains per month during the baseline and the MovEN-intervention in study I

	Gain per month				*t*(15)	*p*	Cohen’s *d*
	During baseline		During intervention				
	*M*	SD	*M*	SD			
EN total score	0.65	0.49	2.12	1.39	3.573	**0.003**	0.893
EN numerical relation	0.33	0.22	1.39	0.98	3.608	**0.003**	0.902
EN counting	0.31	0.38	0.73	0.94	1.625	0.125	0.406
SYMP	0.29	1.68	2.12	4.52	1.390^a^	0.186	0.359
FMS	0.48	0.78	1.19	1.76	0.951^b^	0.366	0.310

Bolded *p*-values are statistically significant (*p* < 0.05)

^a^*t*(14)

^b^*t*(9)

### Results of Study II

Paired samples *t* test was used to examine the immediate and long-term effects of the MovEN-intervention (Table 8). Children’s total EN performance improved in both the intervention (*t*(8) = 2.697, *p* = 0.027) and the average performance control group (*t*(8) = 2.329, *p* = 0.048) during the intervention and control period. However, the effects were larger in the MovEN-intervention group (large; *d* = 0.899) than in the average performance control group (medium; *d* = 0.776), and the intervention effects were sustained in the delayed post-measurement. Children’s numerical relational and FMS performance improved significantly in the intervention group (*t*(8) = 4.031, *p* = 0.004), while no significant improvements were observed in the average performance control group. The effect of the intervention on EN was sustained in the delayed post-measurement. No significant effects were observed on counting or SYMP performance in either group. At the delayed post-measurement, there were no significant differences in numerical relational performance between the groups *t*(8.770) = 1.32, *p* = 0.056.

**Table 8:** Descriptive statistics and results from study II paired samples *t* test

	Pre		Post 1		Post 2		Pre—Post 1			Post 1—Post 2		
	*M*	SD	*M*	SD	*M*	SD	*t*(8)	*p*	Cohen’s *d*	*t*(8)	*p*	Cohen’s *d*
*EN total*												
Intervention	9.89	5.011	13.22	6.996	13.63	7.050	2.697	**0.027**	0.899	0.447^a^	0.668	0.158
Control	13.78	2.489	16.00	2.828	17.11	2.667	2.329	**0.048**	0.776	1.089	0.308	0.363
*EN numerical relation*												
Intervention	6.44	2.789	9.56	4.126	9.88	4.051	4.031	**0.004**	1.344	0.513^a^	0.623	0.182
Control	10.89	1.537	12.00	2.345	13.00	1.936	1.250	0.247	0.417	1.225	0.256	0.408
*EN counting*												
Intervention	3.44	3.468	3.67	3.606	3.75	3.845	0.347	0.738	0.116	0.284^a^	0.785	0.100
Control	2.89	1.269	4.00	1.323	4.11	2.205	1.971	0.084	0.657	0.121	0.907	0.045
*SYMP*												
Intervention	34.56	9.825	33.44	6.327	35.75	10.964	− 0.506	0.626	− 0.169	0.533^a^	0.610	0.189
Control	31.44	3.678	31.56	7.764	31.67	8.803	0.055	0.957	0.018	0.082	0.937	0.027
*FMS*												
Intervention	19.44	6.126	24.56	8.187	23.86	9.737	3.398	**0.009**	1.133	− 0.289^b^	0.782	− 0.109
Control	20.11	3.983	18.89	7.390	22.22	4.086	− 0.593	0.569	− 0.198	1.925	0.090	0.641

Bolded *p*-values are statistically significant (*p* < 0.05)

^a^*t*(7)

^b^*t*(6)

In order to examine the improvements during the intervention period in the intervention and average performance control group, we compared the gains per month (Table 9). While the gains in total EN, numerical relational and FMS performance were greater during the MovEN-intervention compared to the gains in the average performance control group, the effect was statistically significant only in FMS performance.

**Table 9:** Gains per month during the MovEN-intervention and control period in study II

	Gain per month				*t*(16)	*p*	Cohen’s *d*
	During control period		During intervention				
	*M*	SD	*M*	SD			
EN total score	1.111	1.431	1.667	1.854	0.712	0.487	0.335
EN numerical relation	0.556	1.333	1.556	1.158	1.699	0.109	0.801
EN counting	0.556	0.846	0.111	0.961	− 1.042	0.313	0.491
SYMP	0.056	3.025	− 0.556	3.292	− 0.410	0.687	0.193
FMS	− 0.611	3.090	2.556	2.256	2.483	**0.024**	1.170

Bolded *p*-values are statistically significant (*p* < 0.05)

## Discussion

Both FMS and EN skills start to develop in early childhood (Clements & Sarama, 2009; Gallahue & Ozmun, 2002), and the gap between low-, and high-performers widen during the preschool years (Anders et al., 2012; Starkey & Klein, 2008). This highlights the importance of early interventions to support low-performing children, in order to prevent them from being at risk for later mathematical learning difficulties (Aunio et al., 2015). In the present pilot study, we examined the immediate and long-term effects of the MovEN-intervention program, which aims to improve preschoolers’ EN skills by combining the learning of numerical relational skills with FMS practice. The MovEN-intervention was first conducted with a within-subject repeated-measures design (study I) and after some modifications with a quasi-experimental design (study II). By conducting the intervention with two intervention designs, it was possible to increase the reliability of the results despite the small sample sizes.

Both studies demonstrated that children’s EN skills and especially numerical relational skills improved during the MovEN-intervention. This finding is in line with previous story reading interventions demonstrating positive effects on numerical relational skills (Casey et al., 2008; Hassinger-Das et al., 2015; Purpura et al., 2017; Van den Heuvel-Panhuizen & Elia, 2011) and EN skills (Purpura et al., 2017). When comparing gains per month in study I, EN improved significantly more during the eight-week intervention than during the 5-to-10 month baseline period, which is in line with findings from previous combined EN and FMS interventions (Fischer et al., 2011; Shoval et al., 2018). In study II, the gain per month in numerical relational skills was notably larger (*d* = 0.801), albeit statistically nonsignificant (*p* = 0.109), in the MovEN-intervention group compared to the average performance control group. While these differences did not reach statistical significance, it is important to note that when analyzing small sample sizes, effects that are large in magnitude, yet statistically nonsignificant should not necessarily be disregarded, as the risk of detecting false-negatives is considerable (Sullivan & Feinn, 2012). Importantly, the improvements were sustained in the delayed measurement in both studies. These findings are in accordance with a recent systematic review demonstrating that combining FMS with the learning of cognitive or academic skills offers notable benefits on preschoolers’ cognitive and academic skill development (Jylänki et al., 2022).

In study II, there were significant differences in numerical relational skills between intervention and average performance control group before the intervention. However, the difference between the groups in numerical relational skills was not significant after the intervention, suggesting that the gap between these groups narrowed during the intervention, and the children in the intervention group were able to catch up the average-performing peers during the intervention. According to a review by Mononen et al. (2014), previous EN interventions have been found to support at-risk children’s EN learning; however only one study has demonstrated that the gap between at-risk in EN learning and average performers narrowed during the intervention (Clarke et al., 2011), highlighting the novelty of our findings. This is also important, as there have been contradictory results from previous studies demonstrating that the low-performers did not benefit from a combined FMS and numeracy intervention while the average performers did (Beck et al., 2016).

It is important to note that the sample used in our study included children from heterogeneous socioeconomic and language backgrounds. Previous studies have reported that children with low-SES and second language learner status tend to have lower language skills (Raviv et al., 2004; Smith & Dixon, 1995). Indeed, the results of study II demonstrated that children with second language learner status had low-SES, as well as lower language skills. This is noteworthy as numerical relational skills can be more challenging for children with lower language skills, since children are required to possess adequate language skills to understand the linguistically expressed concepts (Purpura & Lonigan, 2013). Nonetheless, in our study, we demonstrated that regardless of the SES or language background, children benefited from the MovEN-intervention; especially in terms of their numerical relational skills. As there was a relationship between language and EN skills at the baseline, it is possible that in addition to EN skills, children’s language skills improved during the intervention resulting in improvements in the ENT. Thus, in future studies, children’s language skills should be measured also after the intervention, in order to find if the numerical relational skill practicing also supports children’s language skills.

One potential explanation for the efficacy of the MovEN-intervention on children’s EN learning is the comprehensive skill practice via story reading and body movement. Indeed, recommendations for improving EN in the early years include exploring EN through different contexts such as storybooks and combining EN with other curriculum areas (Education Endowment Foundation, 2020). Children can experience EN in a meaningful and informal way via story reading (Van den Heuvel-Panhuizen & Elia, 2011), and it offers children the opportunity to understand and apply the numerical relational concepts (Purpura et al., 2017). Furthermore, in the MovEN-intervention, children are encouraged to extend the numerical relational skills to other daily life contexts (e.g., games in the playground and sport activities). With improved numerical relational skills, children are more likely to be able to engage in EN activities and discussions that further enhance their mathematical knowledge (Purpura et al., 2017), which might explain the sustained effects of the intervention.

MovEN-intervention included features that have been found to be effective in FMS and cognitive skill interventions in children (Pesce et al., 2021). These include face-to-face instruction by trained teachers, a group-based setting in children’s respective preschools, and a focus on skill development rather than exercising itself (Pesce et al., 2021). Based on the logbooks and weekly discussions with the teachers, the intervention—in terms of duration and frequency—was considered as feasible to implement into a preschool schedule in both studies. Furthermore, most of the intervention exercises were rated by the teachers as either ‘excellent’ or ‘good’, and both the children and teachers reported that they enjoyed participating in the intervention. Indeed, one study that combined physical activity with EN (Mavilidi et al., 2018) reported that children enjoyed combined interventions the most, and this could have a positive impact on the results of the intervention. Finally, some suggestions were made to further improve the MovEN-intervention (e.g., change the order of the books), which will be considered for future studies.

Although both studies demonstrated that the MovEN-intervention program can be effective on four-year-olds’ EN and FMS skills, it was not possible to conclude whether practising FMS and EN together is more effective than practising both skills separately. Thus the MovEN-intervention program should be studied further with an intervention-control design including EN only, FMS only and combined EN and FMS intervention groups, as well as a business-as-usual control group. None of the previous combined FMS and EN interventions (Beck et al., 2016; Fischer et al., 2011; Shoval et al., 2018; St. Laurent et al., 2018) have compared the intervention to both EN and FMS learning alone. One study (Fischer et al., 2011) compared a combined FMS and EN intervention to an EN only intervention and one study (Beck et al., 2016) to a FMS only intervention. In both studies, the combined FMS and EN intervention appeared to be more effective than either aspect alone, suggesting the superiority of the combined FMS and EN learning.
